# Evaluation of the choroidal structural and vasculature changes following COVID-19 infection and vaccination using optical coherence tomography angiography

**DOI:** 10.1186/s40942-026-00799-1

**Published:** 2026-02-12

**Authors:** Moataz E. Abdelkader, Mansour H. Ahmed, Mahmoud A. Sultan, Marwa O. Elgendy, Ahmed R. N. Ibrahim, Safaa A. M. Aboud

**Affiliations:** 1 Ophthalmology Department, Faculty of Medicine, Misr University, Cairo, Egypt; 2 Ophthalmology Department, Faculty of Medicine, Beni-Suef University, Beni-Suef, Egypt; 3 Department of Clinical Pharmacy, Beni-Suef University Hospitals, Faculty of Medicine, Beni-Suef University, Beni-Suef, Egypt; 4 Department of Clinical Pharmacy, Faculty of Pharmacy, Nahda University (NUB), Beni-Suef, Egypt; 5 Department of Clinical Pharmacy, College of Pharmacy, King khalid University, Abha, Saudi Arabia

**Keywords:** COVID-19, COVID-19 vaccines, OCTA, Choroidal thickness, Choroidal vasculature, FAZ

## Abstract

**Objective:**

To investigate the impact of COVID-19 infection and various COVID-19 vaccines on choroidal structure, vasculature, and the foveal avascular zone (FAZ) using optical coherence tomography angiography (OCTA).

**Methods:**

This prospective study included 200 participants, divided equally into five groups: 40 healthy controls, 40 individuals one month after COVID-19 infection, and three groups vaccinated with AstraZeneca, Pfizer, or Sinovac. OCTA was performed to measure choroidal thickness (CT), choriocapillaris thickness (CCT), choroidal vessel density (CVD), choriocapillaris vessel density (CC-VD), and FAZ parameters.

**Results:**

There were no significant differences among the five groups in central or mean CT (*p* = 0.246 and *p* = 0.053, respectively). However, significant differences were observed in both central and mean CCT (*p* < 0.001 and *p* = 0.006, respectively). Central and mean CVD also varied significantly among the groups (*p* = 0.004 and *p* = 0.035, respectively), with central CVD being significantly higher in the COVID-19 group than in the AstraZeneca and Pfizer groups (*p* = 0.034 and *p* = 0.03, respectively). Mean CC-VD differed significantly among all groups (*p* = 0.006), showing the highest values in the control group and the lowest in the COVID-19 group. No significant differences were found regarding FAZ area or perimeter (*p* = 0.174 and *p* = 0.334, respectively). However, FAZ circularity was significantly reduced in the COVID-19 group compared to the control and Pfizer groups (*p* = 0.008 and *p* = 0.004, respectively).

**Conclusion:**

COVID-19 infection and certain vaccine types may cause subtle but detectable changes in the choroidal microvasculature. These findings demonstrate the sensitivity of OCTA in identifying microvascular alterations in the posterior segment and enhance understanding of how different COVID-19 vaccines may influence ocular microcirculation.

**Supplementary Information:**

The online version contains supplementary material available at 10.1186/s40942-026-00799-1.

## Introduction

The global outbreak of coronavirus disease 2019 (COVID-19), caused by severe acute respiratory syndrome coronavirus 2 (SARS-CoV-2), has had far-reaching impacts on public health since its identification in December 2019 in Wuhan, China [1–4]. Beyond respiratory symptoms, COVID-19 has been shown to involve multiple organ systems [5–9], including the eyes. Ocular involvement may be direct via angiotensin-converting enzyme 2 (ACE2) receptors, widely expressed in the retina and choroid, or indirect through inflammatory and thrombotic sequelae [10–12].

Multiple studies have reported ocular findings in patients with COVID-19, including conjunctivitis, cotton wool spots, retinal hemorrhages, and microvascular changes. In parallel, increasing attention has been directed toward the ocular effects of COVID-19 vaccination [5–8]. Rare reports of choroiditis, uveitis, and other immune-mediated ocular events have been documented following administration of mRNA or inactivated vaccines [12–15]. Ocular adverse effects after COVID-19 vaccinations have emerged include: facial nerve palsy, abducent nerve palsy, Optic neuritis, Central retinal vein occlusion, acute macular neuroretinopathy, central serous retinopathy, uveitis, multiple evanescent white dot syndrome, Vogt-Koyanagi-Harada disease reactivation, and new-onset Graves’ Disease [16, 17].

Optical coherence tomography angiography (OCTA) is a non-invasive imaging modality capable of detecting microvascular alterations in the retina and choroid. Using OCTA, recent studies have identified changes in choroidal thickness(CT), choroidal vessel density (CVD), and foveal avascular zone (FAZ) morphology in individuals recovering from COVID-19 infection or following vaccination [18, 19].

The aim of this study was to assess changes in CT, CVD and FAZ using OCTA in individuals who have either recovered from COVID-19,or received different COVID-19 vaccines.

## Materials and methods

This prospective cross-sectional observational study was conducted at the Ophthalmic Investigative Unit, Faculty of Medicine, Beni-Suef University, Beni-Suef, Egypt, from March 2022 to March 2023. It included 200 individuals divided into five groups (each with 40 participants, 80 eyes): COVID-19 group: laboratory-confirmed (PCR-positive), unvaccinated cases imaged one month after diagnosis (mild severity: non-hospitalized, no oxygen requirement, assessed by internal medicine); AstraZeneca group: subjects vaccinated with AstraZeneca (adenovirus vector vaccine); Pfizer group: subjects vaccinated with Pfizer-BioNTech (mRNA vaccine); Sinovac group: subjects vaccinated with Sinovac (inactivated virus vaccine); Control group: healthy subjects. (Participants were consecutively recruited from the outpatient clinic. Vaccine groups had no prior SARS-CoV-2 infection verified by negative anti-SARS-CoV-2 IgG antibody testing and PCR at recruitment. Controls had negative IgG/PCR and no vaccination history.)

Subjects with associated systemic or ocular diseases, previous ocular surgery or trauma, high refractive error (± 4 diopters), and history of anticoagulant therapy or other drugs were excluded from the study. Note: Axial length was not measured or adjusted for, despite its potential confounding effect on choroidal metrics. No baseline (pre-infection/pre-vaccination) OCTA was obtained.

All subjects were clinically assessed by internal medicine residents in the out-patient clinic, including vital signs and random blood glucose levels. A detailed history taking, and complete ophthalmic examination including BCVA expressed in decimals using Snellen chart, intraocular pressure (IOP) using Air-puff tonometer CT-80, slit lamp examination, color vision testing using Ishihara charts, and dilated fundus examination using indirect ophthalmoscope, were performed for all participants. No clinically evident fundoscopic abnormalities were observed.

OCTA imaging was performed one month after laboratory-confirmed COVID-19 diagnosis (via PCR) or completion of the full vaccination series. This interval was selected to capture potential subacute microvascular changes while minimizing long-term confounding factors, consistent with prior OCTA studies of post-COVID ocular effects.

OCTA was performed using Topcon 3D-DRI OCT Triton (Topcon Corporation, Tokyo, Japan; Frequency 50–60 Hz). Parameters assessed included subfoveal choroidal thickness (CT), choriocapillaris thickness (CCT), choroidal vessel density (CVD), choriocapillaris vessel density (CC-VD), and FAZ parameters (area, perimeter, circularity). All vaccinated groups were evaluated 1 month after administration of complete doses of the vaccine.

All imaging procedures were performed on the same day of examination. The subjects were imaged using a digital retinal imaging Triton wide-field Swept-Source-Source OCT (WFSS-OCT), Topcon Corporation, Tokyo, Japan, with a central wavelength of 1,050 nm, a scanning speed of 100,000 A-scans per second, and axial and transverse resolutions of 8 μm and 20 μm, respectively, in tissue. Software used for OCT is Fastmap and for OCT-A is IMAGEnet6. All SS-OCT and SS-OCTA images were acquired using radial 3 × 3 and 6 × 6 mm scanning centered on the fovea, with a scan resolution of 1024 xx 12 pixels and repeated three times by the same well-trained examiner. Only images with a signal strength index above 55 were accepted. Any image with a significant motion artifact or a double-vessel pattern was excluded.

A linear B-scan centered on the fovea was performed in all participants. The choroidal thickness (CT) was measured from Bruch’s membrane to the choroidal-scleral interface (CSI) automatically in five separate locations, including the sub-fovea, 500 and 1000 microns from the center of the fovea in the nasal and temporal directions (supplementary Fig. 1).

OCTA imaging was performed on the macular region with a scan of 3 mm x 3 mm and 6 mm x 6 mm field of view. Choroid, choriocapillaris, and FAZ, were measured using the automatic layer segmentation software IMAGEnet provided by the device. Vessel density (VD), expressed as %, and FAZ parameters including FAZ area expressed in mm², FAZ perimeter expressed in mm, and FAZ circularity were measured in each participant. Vessel density was provided automatically by the IMAGEnet software. VD is inferred from the decorrelation motion contrast signal provided by OCTA.

In order to quantify and compare choriocapillaris VD (CC-VD) and choroidal VD (CVD), the instrument automatically applies an Early Treatment Diabetic Retinopathy Study (ETDRS) grid overlaying with an external ring of 2.5 mm of diameter and a central area of 1 mm of diameter. The external ring is composed of superior, inferior, nasal, and temporal quadrants. Total VD was the average of the five ETDRS subfields. In order to measure the CC-VD and CVD, we used the automated algorithm to generate en face OCT choroidal slabs. We defined choriocapillaris as the slab between 0 μm at the Bruch’s membrane (BM) and 20.8 μm under the BM. CVD included the slab between 0 μm at the BM and up to the subfoveal CT that was taken previously from each patient separately, marked by the CSI. These choroidal slabs provide a good visualization of the large choroidal vessels (supplementary Fig. 2).

Both eyes of each participant were included, acknowledging potential inter-eye correlation as a limitation.

### Statistical analysis

Data were collected, coded, and entered into an Excel sheet. Data were analyzed using the Statistical Package for the Social Sciences (SPSS) version 20 (SPSS Inc., Chicago, Illinois, USA) and then processed and tabulated. One-way ANOVA (or Kruskal-Wallis if non-normal) was used for multi-group comparisons of continuous variables, followed by post-hoc Tukey or Dunn’s test with Bonferroni correction to address multiple comparisons across five groups and parameters, controlling type I error. Student t-test was retained for pairwise comparisons only where appropriate. Differences between nominal variables were calculated using the Chi-square test and expressed as frequencies. Correlations between continuous variables were calculated using the Pearson correlation coefficient test. Results are considered significant at p-values less than 0.05.

## Results

The mean age of our participants was 32.08 ± 11.51 (18–60) years, 52% of them were females. The mean BCVA was 0.95 ± 0.06 (0.8-1) and the mean IOP was 15.91 ± 2.28 (10–20) mmHg. The 5 study groups were matched for age and sex. Also, there were no significant differences between the 5 groups as regarding BCVA and IOP (p-value > 0.05) (Table 1).

**Table 1:** Demographic data and ophthalmological examination of the study groups

		Control group		AstraZenca group		Pfizer group		Sinovac group		Post Covid − 19 group		*P* value
		Count	%	Count	%	Count	%	Count	%	Count	%	
Sex	M	16	40.0%	19	47.5%	16	40.0%	23	57.5%	21	52.5%	0.43
	F	24	60.0%	21	52.5%	24	60.0%	17	42.5%	19	47.5%	
		**Mean ± SD**		**Mean ± SD**		**Mean ± SD**		**Mean ± SD**		**Mean ± SD**		
Age		32.42.05 ± 9.06		33.95 ± 11.14		34.35 ± 14.55		33.98 ± 7.52		35.70 ± 11.99		0.2
BCVA		0.96 ± 0.06		0.95 ± 0.06		0.95 ± 0.07		0.95 ± 0.06		0.96 ± 0.06		0.76
IOP		15.98 ± 2.36		15.89 ± 2.32		15.79 ± 2.23		15.83 ± 2.32		16.10 ± 2.23		0.91

BCVA: Best corrected visual acuity

IOP: Intra ocular pressure

Values are expressed as (mean ± SD). *p* < 0.05 is considered significant

**Fig. 1:**
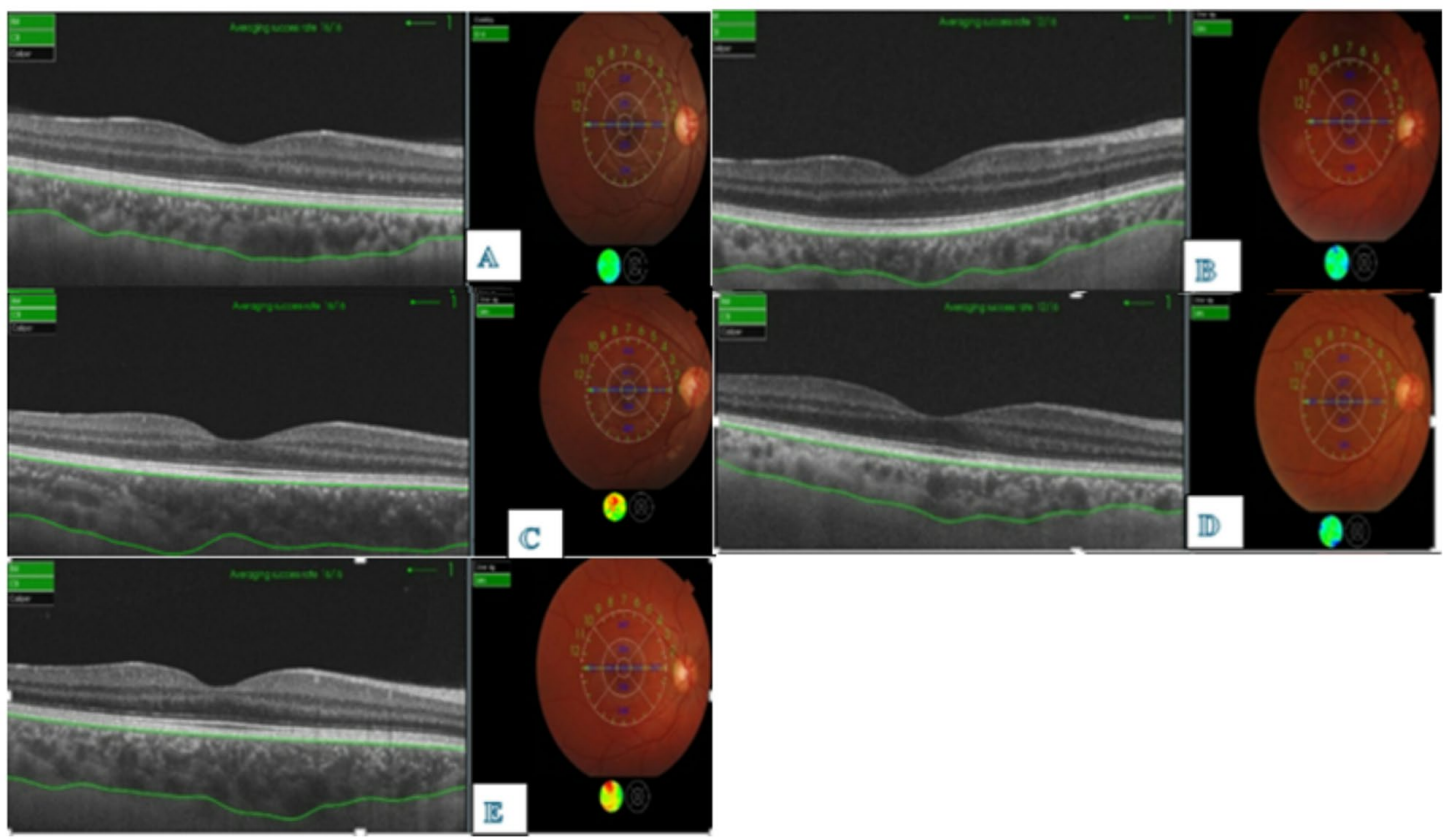
OCT images of Choroidal Thickness in different groups (**A**: Control group, **B**: Astrazeneca group, **C**: Pfizer group, **D**: SinoVac group, **E**: post COVID-19)

Regarding choroidal thickness (CT), there were no significant differences between the 5 groups as regarding sub-foveal(central) and mean CT (*p* = 0.246, *p* = 0.053 respectively, Table 2).However, the AstraZeneca group tended to show the highest CT, while the Pfizer group exhibited the lowest. When comparing the post COVID-19 group to the control group, CT was slightly higher in the former, though the difference remained statistically insignificant (Table 2. Figure 1).

**Table 2:** Comparison between the study groups as regarding choroidal thickness and choriocapillaris thickness

	Thickness(µm)	Control group	AstraZenca group	Pfizer group	Sinovac group	Post COVID-19 group	*P* value
ChoroidalThickness (Mm)	Central	264.62 ± 54.07	279.11 ± 70.40	259.63 ± 66.21	268.10 ± 51.14	271.30 ± 28.96	0.246
	Mean	248.72 ± 46.66	265.86 ± 65.04	243.34 ± 62.39	247.30 ± 46.61	257.08 ± 24.46	0.053
choriocapillaris thickness	Central	52.37 ± 3.15	51.05 ± 3.64	50.96 ± 3.62	53.03 ± 2.99	51.46 ± 4.00	< 0.001
	Mean	52.32 ± 1.47	51.87 ± 1.19	51.88 ± 1.76	52.31 ± 1.34	51.63 ± 1.27	0.006

Values are expressed as (mean ± SD). *p* < 0.05 is considered significant

As regard choriocapillaris thickness (CCT), There was a significant difference among the 5 groups in the subfoveal, and mean thickness (*p* < 0.001,*p* = 0.006 respectively, Table 2). The central CCT was significantly higher in the Sinovac group compared to AstraZenca, Pfizer, and COVID-19 groups (*p* = 0.004, *p* = 0.002, and *p* = 0.047 respectively), without significant differences between other groups. The mean CCT was significantly lower in post COVID-19 group compared to Sinovac group (*p* = 0.026: Supplementary Table 1).

**Fig. 2:**
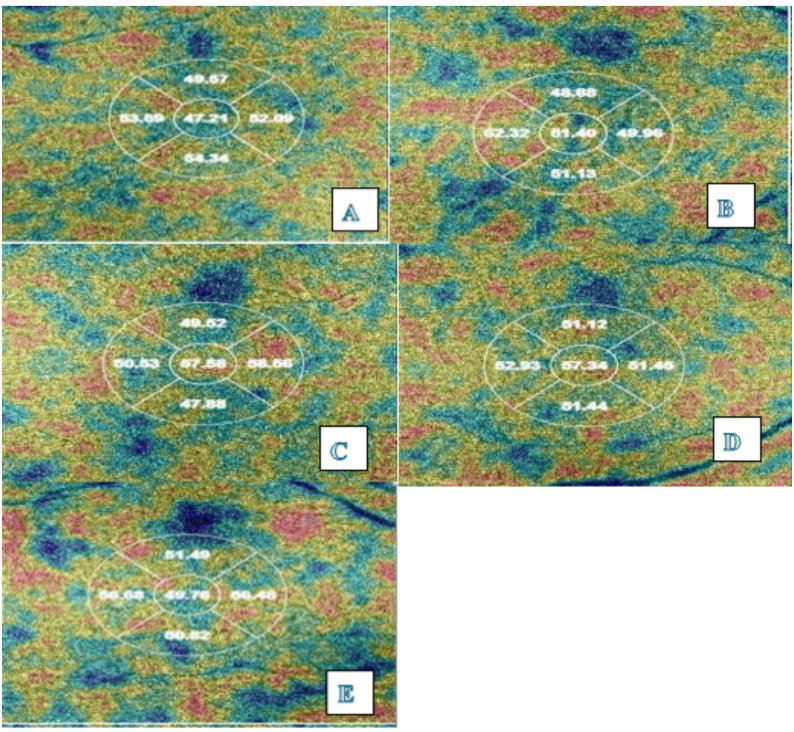
OCT images of Choroidal vessel density in different groups (**A** Control group, **B**: Astrazeneca group, **C**: Pfizer group, **D**: SinoVac group, **E**: post COVID-19)

Regarding choroidal vessel density(CVD), There was a significant difference among the 5 groups in the central, and mean VD (*p* = 0.004, *p* = 0.035 respectively, Table 3; Fig. 2).The central VD was significantly higher in the post COVID-19 group compared to AstraZeneca and Pfizer groups (*p* = 0.034, *p* = 0.03 respectively; Supplementary Table 2).The mean VD was significantly lower in post COVID-19 than Sinovac group (*p* = 0.02; Supplementary Table 2).

**Table 3:** Comparison between the study groups as regarding vessel density and FAZ

VD		Control group	Astrazenca group	Pfizer group	Sinovac group	Post COVID-19 group	*P* value
CVD	Central	53.59 ± 4.04	53.14 ± 3.98	53.12 ± 4.29	54.67 ± 3.43	55.03 ± 4.44	0.004
	mean	52.40 ± 1.51	52.22 ± 1.36	52.28 ± 1.78	52.75 ± 1.35	51.98 ± 1.69	0.035
CC-VD	mean	52.32 ± 1.47	51.87 ± 1.19	51.88 ± 1.76	52.31 ± 1.34	51.63 ± 1.27	0.006
FAZ (mm)	FAZ-Area mm²	0.35 ± 0.11	0.32 ± 0.09	0.34 ± 0.10	0.33 ± 0.12	0.32 ± 0.06	0.174
	FAZ-Perimeter mm	2.58 ± 0.37	2.49 ± 0.40	2.53 ± 0.34	2.52 ± 0.42	2.59 ± 0.26	0.334
	FAZ-Circularity π	0.64 ± 0.06	0.63 ± 0.08	0.65 ± 0.07	0.62 ± 0.08	0.60 ± 0.08	0.002

VD: Vessel density

CVD: Choroidal vessel density

CC-VD: Choriocapillaris vessel density

FAZ: Foveal avascular zone

Values are expressed as (mean ± SD). *p* < 0.05 is considered significant

As regard mean CC-VD, There was a significant difference among the 5 groups where it was the highest in the control group and the lowest in the post COVID-19 group. (*p* = 0.006, Table 3, supplementary Fig. 1).It was signifcantly lower in the post COVID 19 group than both control and Sinovac groups (*p* = 0.023, *p* = 0.026 respectively, Supplementary Table 2).

Comparing the FAZ between the study groups we found no significant differences between the study groups as regarding FAZ-Area and FAZ-Perimeter (*p* = 00.174,*p* = 0.334,Table 3). FAZ-Circularity was significantly lower in the post COVID-19 group compared to control, and Pfizer groups (*p* = 0.008, *p* = 0.004 respectively; Supplementary Table 3).

## Discussion

Our prospective cross-sectional study of 200 participants (400 eyes) identified subtle differences in choroidal microstructure and microvasculature one month following COVID-19 infection or vaccination, detectable by swept-source OCTA despite no clinically evident fundoscopic abnormalities. The groups were well-matched for age, sex, BCVA, and IOP. These OCTA findings highlight the modality’s sensitivity for subclinical posterior segment changes.

In terms of mean choroidal thickness (CT), no statistically significant between-group differences were observed (*p* = 0.246 central, *p* = 0.053 mean). However, the AstraZeneca group showed numerically highest CT values, while the Pfizer group exhibited the lowest. Post-COVID-19 CT was slightly higher than controls but remained statistically insignificant.

The non-significant CT elevation in AstraZeneca recipients may reflect a subclinical vascular response to vaccination[20]. Case reports of ocular inflammatory events such as choroiditis and uveitis following COVID-19 vaccination further support this hypothesis of vaccine-related immune activation affecting the choroid [21, 22].

Similarly, the lower CT in the Pfizer group aligns with a biphasic response reported by Sarta et al., who observed initial subfoveal choroidal thickening followed by a significant reduction below baseline by the fourth week post-Pfizer vaccination [19]. Gedik et al. also demonstrated early post-vaccination reductions in choriocapillaris and deep capillary plexus vessel density, suggesting possible endothelial dysfunction contributing to thinner CT [23]. These patterns are further supported by rare ocular events such as polypoidal choroidal vasculopathy exacerbation and central serous chorioretinopathy following mRNA vaccination, underscoring the choroid’s sensitivity to immune-mediated vascular changes [24, 25]. No CT changes were found post-Sinovac vaccination in comparable timing studies [19]. These associations suggest differential vaccine platform effects on choroidal structure, warranting longitudinal confirmation [26].

Our findings, therefore, suggest a possible transient impact of mRNA vaccination on choroidal thickness, mediated through cytokine-driven vascular modulation or perfusion changes. Future longitudinal studies with serial OCT and OCT-A measurements are needed to clarify these mechanisms.

Regarding COVID-19 infection itself, previous literature has presented inconsistent results. Boyraz et al. reported significantly increased CT in patients during the active phase of COVID-19, which normalized by the third month [27]. Ozturk et al. noted a trend toward increased CT four months post-infection, though values remained lower than controls [28]. Zor et al. observed a significant early thickening of the choroid in non-hospitalized patients, suggesting a peak immune response followed by a reduction [29].

This supports our findings, as imaging was performed within one month post-infection. Other studies such as Abrishami et al., Yorgun et al., Gül and Timurkaan, Cetinkaya et al., and Firat and Kobat all reported no significant differences in CT between COVID-19 and control groups across various post-infectious intervals [26, 30]. Conversely, Keles observed lower CT in COVID-19 patients (18–120 days post-infection), although not statistically significant [31]. Hepokur et al. observed an early decrease in CT post-infection, which normalized by 9 months [32]. Beyoğlu et al. found significantly lower CT in COVID-19 patients, attributing it to vascular damage [21]. Erdem et al. also observed decreased CT in COVID-19 cases and linked it to hypoxia-related cytokine-induced edema and perfusion decline [33]. Sumer and Subaşı similarly reported significant CT reduction over 6 months post-infection, associated with decreased deep capillary and choriocapillaris flow [34].

Regarding choroidal vascular density (CVD), vessel density (VD) in the choroidal layers plays a critical role in the subfoveal blood flow and, by extension, visual function. Prior studies have emphasized the importance of choroidal blood supply in maintaining retinal health [35]. Moreover, CVD measured via OCTA has emerged as a valuable tool for assessing choroidal vascular status, with VD recognized as essential for evaluating microcirculation [36, 37].

In our study, we hypothesized that vascular dysregulation following COVID-19 infection or vaccination could lead to alterations in choroidal perfusion. Thus, we examined both CVD and choriocapillaris vessel density (CC-VD) to assess their potential involvement in post-infectious or post-vaccination ocular microcirculatory changes. Notably, this study is among the first to use wide-field OCTA to evaluate topographic CVD in both COVID-19-recovered and vaccinated individuals.

Our findings showed that, the central CVD was significantly higher in post COVID 19 group compared to AstraZeneca group and Pfizer group, and the mean CVD was highest in the Sinovac group and lowest in the COVID-19 group, with a statistically significant difference between these two groups. While literature on CVD specifically remains limited, most OCTA studies have reported reduced retinal VD in COVID-19 patients, supporting the concept of COVID-19-induced microvasculopathy [30, 38, 39]. The elevated mean CVD in the Sinovac group may be related to the vaccine’s immunogenic profile. Inactivated virus vaccines like Sinovac trigger a strong humoral response but comparatively weaker cellular immunity than mRNA or viral vector platforms [40, 41]. This immune imbalance may lead to prolonged subclinical inflammation that could increase choroidal vascular perfusion [42, 43]. Additionally, persistent antigenic stimulation may induce subtle endothelial dysfunction [44]. This enhanced vasculature may also represent a compensatory response to systemic immune activation [33].

Conversely, the significantly reduced CVD in the COVID-19 group may reflect direct endothelial injury caused by SARS-CoV-2 via angiotensin converting enzyme 2 (ACE2) receptor binding, triggering thromboinflammatory events and reduced perfusion [45]. Chronic inflammation may also lead to choroidal thinning and vascular dropout, consistent with post-viral syndromes [46].

As regard to mean CC-VD, it was highest in the control group and lowest in the COVID-19 group, with a significant difference between both control and the Sinovac groups versus COVID-19 groups. The preservation of CC-VD in healthy individuals underscores the importance of intact endothelial integrity and absence of systemic inflammation [47].Reduced CC-VD in COVID-19 cases is likely due to direct endothelial damage and microvascular thrombosis [45]. Chiosi et al. reported significantly lower CC-VD in recovered COVID-19 patients, particularly in those previously hospitalized, suggesting microangiopathy in the subfoveal region [48]. Kal et al. also noted persistent CC-VD reduction even six months post-infection, attributing this to endothelial injury via ACE2-mediated mechanisms [49].Demir et al., on the other hand, found increased CC flow in COVID-19 patients, which they interpreted as a reactive vasodilation due to hypoxia-induced ischemia of the outer retinal layers [50]. Because the choroid lacks the autoregulation mechanisms of the retina, it is more vulnerable to systemic circulatory changes [47].

Interestingly, both CVD and CC-VD followed similar trends in our study, especially between the COVID-19 and Sinovac groups. This suggests a possible link between choroidal vascular status and immune response type. Further longitudinal research is necessary to understand these mechanisms and their clinical significance.

Foveal Avascular Zone (FAZ) analysis in our study revealed no statistically significant differences in mean area or perimeter. However, FAZ circularity FAZ-Circularity was significantly lower in Covid-19 group compared to control, and Pfizer groups. FAZ circularity was highest in healthy controls and lowest in the COVID-19 group among all groups, indicating greater perifoveal irregularity in COVID individuals. Notably, Pfizer-vaccinated participants maintained FAZ circularity comparable to healthy controls. The disrupted circularity in the COVID-19 group may result from hypoxia, ischemia, and thromboinflammation affecting perifoveal capillaries similar to mechanisms seen in diabetic retinopathy [51]. The literature remains inconsistent regarding FAZ alterations following COVID-19.Conversely, the preserved circularity in the Pfizer group aligns with studies suggesting mRNA vaccines may limit systemic vascular injury [52]. Similar to ours, Bicket et al., Abrishami et al., and Kazantzis et al., all reported no significant changes in FAZ area compared to controls [18, 30, 53]. Meta-analysis by Kazantzis et al., involving 12 studies, revealed no significant difference in FAZ perimeter between COVID-19 patients and controls, supporting our finding that FAZ perimeter followed FAZ area trends without significant change [54]. In contrast, some researchers reported FAZ enlargement post-COVID. Yildiz et al. found significantly larger FAZ area in recovered patients, though perimeter changes were not significant [55]. Portilho et al. and Güemes Villahoz et al. also noted similar FAZ increases, with a limited statistical significance [56, 57]. Zapata et al. observed enlarged FAZ area and reduced vessel density in post-COVID-19 cases, suggesting microvascular ischemia, although they did not evaluate FAZ circularity or compare findings across vaccine groups [58]. Zhang et al. found FAZ stability one week and two months post-recovery implying transient vascular effects in mild cases, other studies offer mixed results [59]. These vascular alterations [60] may serve as early biomarkers of macular ischemia [61–64].

Such conflicting findings may stem from variability in disease severity, timing of imaging, comorbidities, and the accuracy of self-reported infection history in control subjects [65]. Prospective studies with laboratory-confirmed infection status are ideal but logistically difficult, especially during pandemics. Our study attempted to address these limitations by including only unvaccinated, laboratory-confirmed cases and minimizing confounders.

Importantly, our findings one month post-infection are consistent with the longitudinal studies of Abrishami et al. and Zhang et al., both of which documented stable FAZ values after COVID-19 recovery [22, 37]. Zhang further hypothesized a temporary increase in retinal blood flow during early recovery, which normalizes within two months [7] This may reflect a physiological autoregulatory compensation to inflammation or hypoxia. When this mechanism fails, retinal perfusion deficits, such as FAZ enlargement or reduced vessel density may emerge [47, 49, 66].

Regarding vaccination, Salah El-Dein et al. found no significant change in FAZ area one week after various COVID-19 vaccines [67]. Similarly, Gedik et al. and Yorgun et al. reported no FAZ alteration after Sinovac vaccination [15, 18]. Collectively, these findings suggest minimal short-term impact of inactivated vaccines on FAZ morphology.

### Study limitations

- Cross-sectional design without baseline (pre-exposure) OCTA prevents causal attribution; differences may reflect pre-existing traits rather than exposure effects.
- Axial length unmeasured/unadjusted, despite confounding choroidal metrics.
- COVID-19 group limited to mild, non-hospitalized cases from outpatient recruitment; no severity/hospitalization stratification.
- Both eyes analyzed (*n* = 80/group), introducing inter-eye correlation that may inflate significance; one eye/participant preferred in future designs.
- Modest sample size (*n* = 40/group) and single timepoint limit generalizability.

## Conclusions

This cross-sectional study demonstrates associations between COVID-19 infection/certain vaccines and subtle choroidal microvascular differences detectable by OCTA, in the absence of clinically evident fundoscopic abnormalities. These findings highlight OCTA’s sensitivity as a non-invasive biomarker for posterior segment microvasculature. Longitudinal studies with baseline imaging, axial length adjustment, and severity stratification are needed to establish causality and clinical significance.

## Supplementary Information

Below is the link to the electronic supplementary material.

Supplementary Material 1

## Data Availability

Raw data supporting this study’s findings are available from the corresponding author upon reasonable request.
