# Supplementary material for: Evaluation of the choroidal structural and vasculature changes following COVID-19 infection and vaccination using optical coherence tomography angiography
Source: Int J Retina Vitreous. 2026 Feb 12;12:36. doi: 10.1186/s40942-026-00799-1 (PMC12914983; doi:10.1186/s40942-026-00799-1)
Supplement: Supplementary file 1 — Supplementary Material 1 [file 40942_2026_799_MOESM1_ESM.docx]

**Supplementary Tables**

**Supplementary table 1:** Post hoc pairwise comparison (P value between each 2 groups)

| **Choriocapillaris thickness (mm)** |  | **AstraZenca group** | **Pfizer group** | **Sinovac group** | **Post-COVID 19 group** |
| --- | --- | --- | --- | --- | --- |
| **central** | **Control group** | 0.176 | 0.112 | 1.000 | 1.000 |
|  | **AstraZenca group** |  | 1.000 | 0.004 | 1.000 |
|  | **Pfizer group** | 1.000 |  | 0.002 | 1.000 |
|  | **Sinovac group** | 0.004 | 0.002 |  | 0.047 |
| **mean** | **Control group** | 0.453 | 0.524 | 1.000 | 0.023 |
|  | **AstraZenca group** |  | 1.000 | 0.492 | 1.000 |
|  | **Pfizer group** | 1.000 |  | 0.567 | 1.000 |
|  | **Sinovac group** | 0.492 | 0.567 |  | 0.026 |

Values are expressed as (mean ± SD). *p* < 0.05 is considered significant.

**Supplementary table 2 :**Post hoc pairwise comparison (P value between each 2 groups)

| Vessel density(VD) per mm |  | **Astrazenca group** | **Pfizer group** | **Sinovac group** | **Post COVID-19 group** |
| --- | --- | --- | --- | --- | --- |
| **Central CVD** | **Control group** | 1.000 | 1.000 | 0.914 | 0.247 |
|  |  |  |  |  |  |
|  | **Astrazenca group** |  | 1.000 | 0.175 | 0.034 |
|  | **Pfizer group** | 1.000 |  | 0.158 | 0.030 |
|  | **Sinovac group** | 0.175 | 0.158 |  | 1.000 |
| **Mean CVD** | **Control group** | 1.000 | 1.000 | 1.000 | 0.870 |
|  | **Astrazenca group** |  | 1.000 | 0.325 | 1.000 |
|  | **Pfizer group** | 1.000 |  | 0.574 | 1.000 |
|  | **Sinovac group** | 0.325 | 0.574 |  | 0.020 |
| **Mean CC-VD** | **Control group** | 0.453 | 0.524 | 1.000 | 0.023 |
|  | **Astrazenca group** |  | 1.000 | 0.492 | 1.000 |
|  | **Pfizer group** | 1.000 |  | 0.567 | 1.000 |
|  | **Sinovac group** | 0.492 | 0.567 |  | 0.026 |

Values are expressed as (mean ± SD). *p* < 0.05 is considered significant.

**Supplementary Table 3 :**Post hoc pairwise comparison (P value between each 2 groups)

|  |  | **Astrazenca group** | **Pfizer group** | **Sinovac group** | **Post COVID-19 group** |
| --- | --- | --- | --- | --- | --- |
| **FAZ-Circularity π** | **Control group** | 1.000 | 1.000 | 0.628 | 0.008 |
|  | **Astrazenca group** |  | 1.000 | 1.000 | 0.130 |
|  | **Pfizer group** | 1.000 |  | 0.421 | 0.004 |
|  | **Sinovac group** | 1.000 | 0.421 |  | 1.000 |

Values are expressed as (mean ± SD). *p* < 0.05 is considered significant.


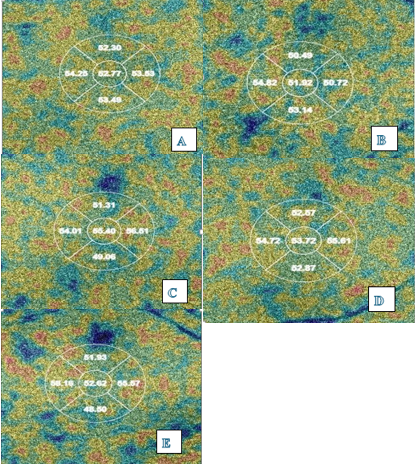


***Supplementary Figure (1):*** OCT images of Choroicapillaris vessel density in different groups (A Control group, B: Astrazeneca group, C: Pfizer group, D: SinoVac group, E: post COVID-19)
